# Enhancement of antinociception by coadminstration of minocycline and a non-steroidal anti-inflammatory drug indomethacin in naïve mice and murine models of LPS-induced thermal hyperalgesia and monoarthritis

**DOI:** 10.1186/1471-2474-11-276

**Published:** 2010-12-01

**Authors:** Ala'a Ahmed Abu-Ghefreh, Willias Masocha

**Affiliations:** 1 Department of Applied Therapeutics, Faculty of Pharmacy, Kuwait University, P.O. Box 24923 Safat, 13110 Kuwait

## Abstract

**Background:**

Minocycline and a non-steroidal anti-inflammatory drug (NSAID) indomethacin, have anti-inflammatory activities and are both used in the management of rheumatoid arthritis. However, there are no reports on whether coadministration of these drugs could potentiate each other's activities in alleviating pain and weight bearing deficits during arthritis.

**Methods:**

LPS was injected to BALB/c mice intraperitoneally (i.p.) to induce thermal hyperalgesia. The hot plate test was used to study thermal nociception in naïve BALB/c and C57BL/6 mice and BALB/c mice with LPS-induced thermal hyperalgesia and to evaluate antinociceptive effects of drugs administered i.p. Monoarthritis was induced by injection of LPS intra-articularly into the right hind (RH) limb ankle joint of C57BL/6 mice. Weight bearing changes and the effect of i.p. drug administration were analyzed in freely moving mice using the video-based CatWalk gait analysis system.

**Results:**

In naïve mice indomethacin (5 to 50 mg/kg) had no significant activity, minocycline (25 to 100 mg/kg) produced hyperalgesia to thermal nociception, however, coadministration of minocycline 50 mg/kg with indomethacin 5 or 10 mg/kg produced significant antinociceptive effects in the hot plate test. A selective inhibitor of COX-1, FR122047 (10 mg/kg) and a selective COX-2 inhibitor, CAY10404 (10 mg/kg) had no significant antinociceptive activities to thermal nociception in naïve mice, however, coadministration of minocycline, with CAY10404 but not FR122047 produced significant antinociceptive effects. In mice with LPS-induced hyperalgesia vehicle, indomethacin (10 mg/kg) or minocycline (50 mg/kg) did not produce significant changes, however, coadministration of minocycline plus indomethacin resulted in antinociceptive activity. LPS-induced RH limb monoarthritis resulted in weight bearing (RH/left hind (LH) limb paw pressure ratios) and RH/LH print area ratios deficits. Treatment with indomethacin (1 mg/kg) or minocycline (50 mg/kg) had no effects on the weight bearing and print area ratios deficits of monoarthritic mice. However, combination of minocycline plus indomethacin restored weight bearing and paw print area ratios of monoarthritic mice similar to that observed in non-arthritic control mice.

**Conclusions:**

Coadministration of indomethacin or a selective COX-2 inhibitor, CAY10404 with minocycline potentiates their effects and results in antinociception against thermal nociception, reduction of thermal hyperalgesia and alleviation of weight bearing deficits in monoarthritic mice at doses where either drug alone has no significant activity. Thus, the coadministration of lower doses of a NSAID or a selective COX-2 inhibitor plus minocycline could be useful in the management of inflammatory pain and arthritis.

## Background

Minocycline, a second-generation semisynthetic tetracycline antibiotic, has pleiotropic biologic activities besides its antimicrobial activities. Apart from its use as an antibiotic it is used in the management of various inflammatory diseases such as rheumatoid arthritis, periodontitis and several dermatological conditions [1]. In the management of rheumatoid arthritis minocycline is used as a disease-modifying antirheumatic drug (DMARD) and it alleviates joint tenderness and swelling among other features of the disease [2-5].

Recently, interest has arisen on its possible use in the management of pain. Research has been mostly on neuropathic pain where it has been shown that preemptive treatment with minocycline has protective effects but is ineffective once pain has developed [6,7]. It has also been reported to have antinociceptive effects in various models of inflammatory pain such as the formalin-induced nociception test and its antinociceptive effects have been suggested to be more of an anti-inflammatory nature rather than a centrally acting analgesic [8].

In this study the antinociceptive effects of co-administration of minocycline with a non-steroidal anti-inflammatory drug (NSAID), indomethacin, a selective inhibitor of COX-1, FR122047 or a selective COX-2 inhibitor, CAY10404, were evaluated in naïve mice and mice with lipopolysaccharide (LPS)-induced thermal hyperalgesia and monoarthritis. LPS is an endotoxin from gram-negative bacterial cell wall, which when administered intra-articularly into the ankle joint of a rodent limb can induce arthritis [9,10]. When the endotoxin is administered intraperitoneally it can cause generalized hyperalgesia. Various mechanisms are purported to be responsible for the LPS-induced hyperalgesia including the release and activation of pro-inflammatory cytokines and gelatinases, as well as glial cell activation [11-15]. Cytokines such as TNF-α and IL-1β cause the release of nociceptive mediators such as prostaglandins and sympathetic amines which lower the nociceptive threshold of sensory nerves [16-19]. Indomethacin is used in the treatment of various inflammatory diseases such as rheumatoid arthritis, osteoarthritis, gout, and ankylosing spondylitis [20-23]. NSAIDs are a heterogenous group of drugs commonly used in the management of inflammatory pain [24-27]. They produce their basic analgesic effect via inhibition of cyclooxygenase (COX) enzymes [25-27]. The analgesic effects of NSAIDs are also due to a number of mechanisms including a central mechanism involving both prostaglandin synthesis inhibition and probably changes in the endocannabinoid and monoaminergic system [28-32].

## Methods

### Animals

BALB/c (n = 338) and C57BL/6 (n = 32) mice (8 to 12 weeks old; 20 - 30 g) were used and were supplied by the breeding unit at the Health Sciences Center, Kuwait University, Kuwait, and were kept in temperature controlled (24 ± 1°C) rooms with food and water *ad libitum*. All experiments were performed during the same period of the day (8:00 AM to 4:00 PM) to exclude diurnal variations in pharmacological effects. The animals were handled in compliance with European Communities Council Directive 86/609 for the care of laboratory animals and ethical guidelines for research in experimental pain with conscious animals [33]. All procedures were approved by the Kuwait University Health Sciences Center animal care committee.

### Drug treatment and assessment of thermal nociception

Minocycline and indomethacin (up to 10 mg/kg) (Sigma-Aldrich, St Louis, MO, USA) were dissolved in phosphate buffered saline (PBS). Indomethacin (≥ 20 mg/kg) (Sigma-Aldrich, USA)), selective COX-1 inhibitor, FR122047 [34,35] and selective COX-2 inhibitor, CAY10404 [36] (both from Cayman Chemical, Ann Arbor, MI, USA) were dissolved in peanut oil (Sigma-Aldrich, USA).

BALB/c or C57BL/6 mice were treated intraperitoneally (i.p.) with minocycline, indomethacin, FR122047, CAY10404 or their vehicles at a volume of 5 μl/g body mass. For the evaluation of coadministration, mice received two separate i.p. injections at the same time: minocycline + indomethacin, minocycline + FR122047, minocycline + CAY10404, minocycline + vehicle for indomethacin, minocycline + vehicle for FR122047, minocycline + vehicle for CAY10404, indomethacin + vehicle for minocycline, FR122047 + vehicle for minocycline or CAY10404 + vehicle for minocycline.

Thermal hyperalgesia was induced by administering LPS (Sigma-Aldrich, St Louis, MO, USA) at 1 mg/kg at a volume of 5 μl/g body mass. Reaction latency to the hot plate were measured before LPS administration and over a period of 1 month after LPS administration. Mice were treated intraperitoneally with drugs on the seventh day after LPS inoculation based on thermal hyperalgesia results (see Results section) and the effects of drug treatment on LPS-induced thermal hyperalgesia evaluated.

Reaction latencies to hot plate test were measured before (baseline latency) and at various times starting at 30 minutes after drug treatment. Briefly, mice were individually placed on a hot plate (Panlab SL, Barcelona, Spain) with the temperature adjusted to 55 ± 1 °C. The time to the first sign of nociception, paw licking, flinching or jump response to avoid the heat was recorded and the animal immediately removed from the hot plate. A cut-off period of 20 seconds was maintained to avoid damage to the paws.

### Induction of monoarthritis and evaluation of changes in weight bearing and paw print area

Inflammatory monoarthritis was induced as described previously [9,10]. Briefly, mice were anaesthetized with halothane and LPS 10 μg in 20 μl PBS was injected intra-articularly into the right hind limb ankle joint through the Achilles tendon using a 30½-gauge needle. PBS (20 μl) was administered in the same way to the control group. The mice were made to cross the Catwalk walkway before LPS-inoculation and at 2 days post-LPS inoculation as described previously [10].

Mice were treated intraperitoneally with indomethacin 1 and 10 mg/kg, minocycline (50 mg/kg) alone, both drugs at the same time or their vehicles on the second day after LPS inoculation based on the weight bearing results [10]. The mice were then made to cross the Catwalk walkway at 1 hour post-drug treatment.

Paw pressure intensity and print areas of freely moving animals were measured using the Catwalk gait analysis system (Noldus Information Technology, The Netherlands) as described previously [10,37]. Briefly, the CatWalk instrument consists of an enclosed walkway with a glass plate, a high speed colour camera, and a recording and analysis software to assess the locomotor performance of rodent models. Each mouse was placed individually in the CatWalk walkway and allowed to walk freely and traverse from one side to the other of the walkway glass plate. The recordings were carried out when the room was completely dark, except for the light from the computer screen. Light from a fluorescent lamp was emitted inside the glass plate and completely internally reflected. Where the mouse paws made contact with the glass plate, light was reflected down and the illuminated contact areas recorded with a high speed colour video camera that was positioned underneath the glass plate connected to a computer that runs the CatWalk software 7.1. The software automatically labelled all the areas containing pixels above the set threshold (7 pixels). These areas were identified and assigned to the respective paws. Analysis of the recording generated a wide range of parameters of which only paw pressure (light intensity, which is the mean brightness of all pixels of the print at maximum paw contact, ranging from 0-255 arbitrary units) and paw print area (complete surface area contacted by the paw during a stance phase) were analysed.

### Data analysis

The results in the text and figures are expressed as the means ± S.E.M. For the hot plate test percentage of change from baseline latency was calculated as follows: for naïve mice (response latency after drug treatment - baseline latency)/baseline latency × 100 and for mice with LPS-induced hyperalgesia (response latency after drug treatment - latency before drug treatment at 7 days post LPS inoculation)/latency before drug treatment at 7 days post LPS inoculation × 100. For the Catwalk gait analysis weight bearing was measured as the right hind (RH)/left hind (LH) limb ratio of the light intensity and the print area ratio was obtained from the RH/LH print areas. Percentage of change after drug treatment was calculated as follows: (light intensity or print area ratio after drug treatment - light intensity or print area ratio before drug treatment at 2 days post LPS inoculation)/light intensity or print area ratio before drug treatment at 2 days post LPS inoculation × 100. Statistical analyses were performed using two-way analysis of variance (ANOVA) followed by Bonferroni post-tests or one-way ANOVA followed by Newman-Keuls multiple comparison test. The differences were considered significant at p < 0.05.

## Results

### Effect of treatment with indomethacin, minocycline or coadministration of minocycline plus indomethacin in a hot plate test in naïve BALB/c and C57BL/6 mice

**Figure 1:**
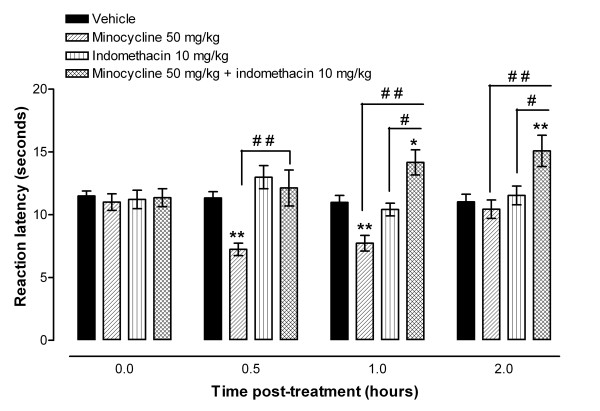
**Coadministration of minocycline with indomethacin produces antinociceptive effects in the hot plate test**. Effect of intraperitoneal treatment with indomethacin, minocycline or a combination of minocycline plus indomethacin in a hot plate test in naïve BALB/c mice. Time course of the reaction latency times for minocycline (50 mg/kg, n = 15), indomethacin (10 mg/kg, n = 16), minocycline + indomethacin (n = 16) or their vehicles (n = 16). Each point represents the mean ± S.E.M of the values obtained from 15 to 16 animals. Statistically significant differences in comparison with drug vehicle: * p < 0.05 and ** *p *< 0.01; and between minocycline or indomethacin and the combination of minocycline + indomethacin treatments: ^# ^*p *< 0.05 and ^# # ^*p *< 0.01 (two-way ANOVA followed by Bonferroni test).

The administration of indomethacin (5 to 50 mg/kg intraperitoneally) to naïve BALB/c mice did not result in a significant change in reaction latency compared to baseline latency in the hot plate at 55°C compared to vehicle treatment (Figure 1 and Table 1). The administration of minocycline 50 mg/kg (this dose has been used previously in other pain studies [6,8]) intraperitoneally to BALB/c mice produced a time dependent reduction in reaction latency in the hot plate at 55°C (Figure 1). The reaction latency fell from a baseline latency of 12.66 ± 0.42 s to 8.31 ± 0.60 s at 1 hour post minocycline administration and by 2 hours it had returned to baseline reaction latency. The minocycline-induced reduction of reaction latency time from baseline value was dose dependent (Table 1). However, the coadministration of indomethacin 5 or 10 mg/kg and minocycline 50 mg/kg resulted in an increase in the reaction latency time compared to vehicle treated animals or indomethacin or minocycline alone (Figure 1 and Table 1). The effects of indomethacin 10 mg/kg, minocycline 50 mg/kg or coadministration of the two drugs on reaction latency was also tested in another mouse strain, C57BL/6, with similar results (Table 2). In order to determine the role of specific COX isoenzyme inhibition on this potentiated antinociceptive effects, minocycline was coadministered with selective COX inhibitors. The administration of a selective inhibitor of COX-1, FR122047 (10 mg/kg intraperitoneally) or a selective inhibitor of COX-2, CAY10404 (10 mg/kg intraperitoneally) to BALB/c did not result in a significant change in reaction latency compared to baseline latency in the hot plate or compared to vehicle treatment (Table 3). The coadministration of minocycline 50 mg/kg and FR122047 10 mg/kg did not significantly change the reduced reaction latency caused by minocycline (*p *> 0.05, Table 3). However, the coadministration of minocycline 50 mg/kg and CAY10404 10 mg/kg resulted in a significant increase in reaction latency (*p *< 0.01) at 1 hour post treatment compared to vehicle or minocycline alone, respectively (Table 3).

**Table 1:** Percentage of change from baseline reaction latency of BALB/c mice at 1 hour post-drug treatment in the hot plate test.

Drug and dose administered to mice	**% Change in reaction latency (mean ± S.E.M of the values obtained from 6 to 16 animals.) **^**#**^	**Statistical significance**^**§**^
Vehicle for indomethacin	-4.64 ± 4.619 (n = 7)	

Indomethacin 5 mg/kg	-10.43 ± 4.11 (n = 7)	ns

Indomethacin 10 mg/kg	-1.02 ± 3.85 (n = 7)	ns

Indomethacin 20 mg/kg	-6.43 ± 5.75 (n = 7)	ns

Indomethacin 50 mg/kg	2.03 ± 10.29 (n = 7)	ns

Vehicle for minocycline	-5.34 ± 2.68 (n = 16)	

Minocycline 12.5 mg/kg	-6.78 ± 3.09 (n = 11)	ns

Minocycline 25 mg/kg	-21.63 ± 2.24 (n = 11)	**

Minocycline 50 mg/kg	-34.16 ± 4.31 (n = 10)	**

Minocycline 100 mg/kg	-34.21 ± 4.08 (n = 8)	**

Vehicles for indomethacin and minocycline	-4.97 ± 2.51 (n = 16)	

Indomethacin 5 + minocycline 50 mg/kg	21.49 ± 4.70 (n = 6)	**

Indomethacin 10 + minocycline 50 mg/kg	24.567 ± 4.05 (n = 16)	**

Indomethacin 10 + minocycline 25 mg/kg	-7.65 ± 4.97 (n = 6)	ns

^# ^(response latency after drug treatment - baseline latency)/baseline latency × 100.

^§^Statistically significant differences in comparison with drug vehicle: ** *p *< 0.01; ns: no statically significant differences in comparison with drug vehicle (one-way ANOVA followed by Newman-Keuls test).

**Table 2:** Percentage of change from baseline reaction latency times of C57BL/6 mice at 1 hour post-drug treatment in the hot plate test.

Drug and dose administered to mice	**% Change in reaction latency (mean ± S.E.M of the values obtained from 7 to 9 animals.) **^**#**^	**Statistical significance**^**§**^
Vehicles for indomethacin and minocycline	-8.62 ± 5.65 (n = 7)	

Indomethacin 10 mg/kg	3.25 ± 6.05 (n = 8)	ns

Minocycline 50 mg/kg	-34.85 ± 3.88 (n = 8)	**

Indomethacin 10 + minocycline 50 mg/kg	32.74 ± 10.85 (n = 9)	**

^# ^(response latency after drug treatment - baseline latency)/baseline latency × 100.

^§^Statistically significant differences in comparison with drug vehicle: ** *p *< 0.01; ns: no statically significant differences in comparison with drug vehicle (one-way ANOVA followed by Newman-Keuls test).

**Table 3:** Percentage of change from baseline reaction latency times of BALBc mice at 1 hour post-drug treatment with minocycline plus selective COX inhibitors in the hot plate test.

Drug and dose administered to mice	**% Change in reaction latency (mean ± S.E.M of the values obtained from 8 to 11 animals.) **^**#**^	**Statistical significance**^**§**^
Vehicles (for COX inhibitors and minocycline)	-5.11 ± 4.05 (n = 10)	

selective COX-1 inhibitor, FR122047 10 mg/kg	4.33 ± 6.81 (n = 11)	ns

selective COX-2 inhibitor, CAY10404 10 mg/kg	12.85 ± 6.29 (n = 8)	ns

Minocycline 50 mg/kg	-26.34 ± 3.35 (n = 9)	**

FR122047 10 + minocycline 50 mg/kg	-11.68 ± 4.61 (n = 11)	ns, nz

CAY10404 10 + minocycline 50 mg/kg	24.22 ± 5.81 (n = 10)	**, # #

^# ^(response latency after drug treatment - baseline latency)/baseline latency × 100.

^§ ^Statistically significant differences in comparison with drug vehicle: ** *p *< 0.01; ns: no statically significant differences in comparison with drug vehicle; statistically significant differences between minocycline alone and the combination of minocycline + selective COX inhibitor treatments: ^# # ^*p *< 0.01; nz: no statically significant differences in comparison with minocycline alone (one-way ANOVA followed by Newman-Keuls test).

### Effect of treatment with indomethacin, minocycline or coadministration of minocycline plus indomethacin in a hot plate test in BALB/c mice with LPS-induced thermal hyperalgesia

**Figure 2:**
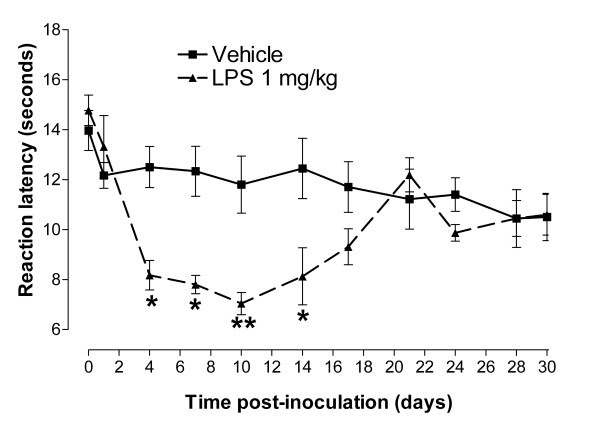
**Lipopolysaccharide (LPS)-induces thermal hyperalgesia in BALB/c mice**. Time course of the reaction latency time to the hot plate test after administration of LPS (1 mg/kg, n = 5, i.p.) or its vehicle (n = 8). Each point represents the mean ± S.E.M of the values obtained from 5-8 animals. Statistically significant differences in comparison with drug vehicle at the same time point post treatment: * p < 0.05 and ** p < 0.01 (two-way ANOVA followed by Bonferroni test).

Inflammatory hyperalgesia to thermal nociception was induced in BALB/c mice by a single i.p. administration of an endotoxin, lipopolysaccharide (LPS, 1 mg/kg), dissolved in PBS. A significant reduction in response latency time to thermal stimuli compared to the baseline latency in the hot plate test was observed in LPS-inoculated mice from 4 to 14 days post inoculation (Figure 2).

**Figure 3:**
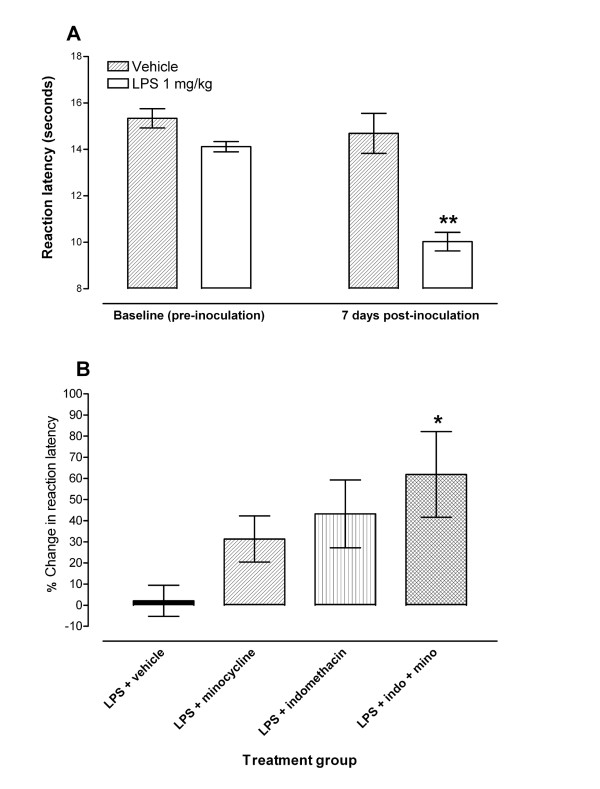
**Coadministration of minocycline with indomethacin enhances their effects and alleviates LPS-induced hyperalgesia**. Effect of treatment with indomethacin, minocycline or a combination of minocycline plus indomethacin on LPS-induced hyperalgesia at day 7 post lipopolysaccharide (LPS, 1 mg/kg, i.p.) inoculation in a hot plate test in BALB/c mice. **A**: Hypernociceptive effects of LPS, 1 mg/kg, i.p. in the hot plate model of inflammatory thermal hyperalgesia in BALB/c mice at 7 days post inoculation. Each point represents the mean ± S.E.M of the values obtained from 6 vehicle-treated and 29 LPS-treated animals. Statistically significant differences in comparison with vehicle inoculated mice at the same time point post treatment: ** p < 0.01 (two-way ANOVA followed by Bonferroni test). **B**: Percentage change in reaction latency times from baseline values (taken at 7 days post LPS) at 1 hour after treatment with minocycline (50 mg/kg, n = 15), indomethacin (10 mg/kg, n = 14), minocycline + indomethacin (n = 16) or their vehicles (n = 12) in hot plate test. Each bar represents the mean ± S.E.M of the values obtained from 12 to 16 animals. Statistically significant differences in comparison with drug vehicle: * p < 0.05 (two-way ANOVA followed by Bonferroni test).

Mice with LPS-induced hyperalgesia to thermal nociception at 7 days post inoculation (Figure 3A) were treated with indomethacin, minocycline or minocycline plus indomethacin coadminstration and their reaction latency to the hot plate recorded.

The administration of indomethacin (10 mg/kg) or minocycline (50 mg/kg) to mice with LPS-induced thermal hyperalgesia did not produce significant changes compared to vehicle treated mice. However, both drugs showed a tendency to have some antinociceptive activity at 1 hour post treatment (increased percentage change in reaction latency after treatment compared to before treatment) in mice with LPS-induced thermal hyperalgesia (Figure 3B). The effects of indomethacin or minocycline compared to vehicle treatment were 31.4 ± 10.9% and 43.2 ± 16.0% increase in reaction latency time after treatment with minocycline or indomethacin, respectively, compared to 2.1 ± 7.4% after vehicle treatment. Coadministration of minocycline 50 mg/kg plus indomethacin 10 mg/kg to mice with LPS-induced hyperalgesia resulted in antinociceptive activity (Figure 3B). The effects of the combination treatment compared to vehicle treatment were significant at 1 hour post treatment (61.9 ± 20.3% in reaction latency time after treatment with minocycline plus indomethacin compared to 2.1 ± 7.4% after vehicle treatment at 1 hour).

### Effect of treatment with indomethacin, minocycline or coadministration of minocycline plus indomethacin in C57BL/6 mice with LPS-induced monoarthritis

Recently we reported that the Catwalk gait analysis system can be used to objectively quantify weight bearing changes and evaluate pharmacological antinociception in freely moving mice with LPS-induced monoarthritis [10].

Weight bearing changes were calculated using the ratio of right hind (RH) limb to the left hind (LH) limb paw pressure intensity (light intensity). The ratio of RH/LH paw print area were also measured.

**Figure 4:**
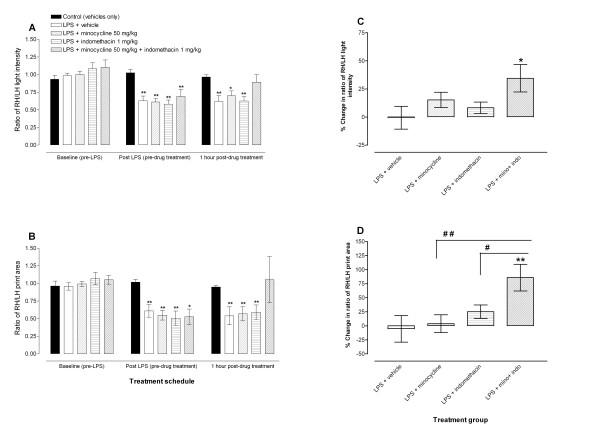
**Coadministration of minocycline with indomethacin alleviates weight bearing deficits in mice with LPS-induced monoarthritis**. Effects of indomethacin 1 mg/kg, minocycline 50 mg/kg or a combination of minocycline 50 mg/kg plus indomethacin 1 mg/kg on **A**: weight bearing (measured as ratio of light intensity between right hind (RH) and left hind (LH) limbs) and **B**: ratio of RH/LH print area of mice with LPS-induced arthritis. The drugs or their vehicles were administered at 2 days post-LPS administration and their effects measured at 1 hour after drug treatment. Each point represents the mean ± S.E.M of the values obtained from 6 to 9 animals. Non-arthritic control (n = 9) and arthritic mice (LPS-inoculated) treated with vehicle (n = 7), indomethacin 1 mg/kg (n = 7), minocycline 50 mg/kg (n = 8) or a combination of minocycline 50 mg/kg plus indomethacin 1 mg/kg (n = 6). Statistically significant differences in comparison with non-arthritic control (vehicle only injected) group: * p < 0.05 and ** p < 0.01 (two-way ANOVA followed by Bonferroni test). **C **and **D**: Percentage change in RH/LH light intensity and print area ratios from LPS-induced monoarthritic mice at 1 hour after drug treatment. Each bar represents the mean ± S.E.M of the values obtained from 6 to 8 animals. Arthritic mice (LPS-inoculated) treated with vehicle (n = 7), indomethacin 1 mg/kg (n = 7), minocycline 50 mg/kg (n = 8) or a combination of minocycline 50 mg/kg plus indomethacin 1 mg/kg (n = 6). Statistically significant differences in comparison with drug vehicle treated group: * p < 0.05 and ** p < 0.01; and between indomethacin or minocycline alone versus minocycline plus indomethacin combination treated mice: ^# ^p < 0.05 and ^# # ^p < 0.01 (one-way ANOVA followed by Newman-Keuls multiple comparison test).

The RH/LH paw pressure ratio of the control group remained constant throughout the experiment, whereas those of LPS-inoculated mice decreased at 2 days after LPS administration compared to baseline or control mice (p < 0.01; Figure 4A) similar to what has been described previously [10].

The changes observed in paw print area ratios in LPS inoculated mice compared to solvent-injected controls were similar to those observed in paw pressure ratios. The RH/LH print area ratio of the control group remained constant throughout the experiment, whereas those of LPS inoculated mice decreased at 2 days after LPS administration compared to baseline or control mice (p < 0.05; Figure 4B).

We observed that a higher dose of indomethacin (10 mg/kg) can alleviate the weight bearing deficits in mice with LPS-induced monoarthritis (data not shown) as previously described [10]. Treatment of mice with LPS-induced monoarthritis using either indomethacin 1 mg/kg or minocycline 50 mg/kg alone had no significant effect on the weight bearing or print area ratios deficits observed in these mice (Figure 4A,B). Mice with LPS-induced monoarthritis which were treated with the combination of minocycline 50 mg/kg plus indomethacin 1 mg/kg had less weight bearing deficits and their RH/LH paw pressure or print area ratios reverted to be similar to control animals, (no significant differences between control mice and those treated with the combination, Figure 4A,B). Coadministration of minocycline 50 mg/kg plus indomethacin 1 mg/kg to mice with LPS-induced monoarthritis significantly increased the RH/LH paw pressure or print area at 1 hour post treatment by 34.5 ± 12.3% and 85.8 ± 23.8% respectively, compared to drug vehicle treated monoarthritic animals which had -0.6 ± 10.1% and -5.4 ± 23.7% change at 1 hour post treatment for RH/LH paw pressure and print area respectively (Figure 4C,D). The effects of coadministration of minocycline plus indomethacin on RH/LH print area ratio was significantly higher than either indomethacin or minocycline treatment (Figure 4D).

## Discussion

This is the first study to report enhanced antinociceptive activity and alleviation of weight bearing deficits by coadministering minocycline plus indomethacin, a NSAID, against thermal and arthritic nociception in mice.

The enhanced antinociceptive effects produced by coadministering minocycline plus indomethacin on inflammatory hyperalgesia and monoarthritis-induced weight bearing deficits could be useful in the management of inflammation and pain in patients with arthritis. Both drugs, minocycline and indomethacin, are used in the management of rheumatoid arthritis (RA) [1,5,21]. However, potentiation of each other's activities by the two compounds in alleviating pain and weight bearing deficits caused by inflammation or arthritis have not yet been reported. This potentiation of analgesic activity could have been missed in human RA because the interest on minocycline has been on its DMARD activity but not analgesic effects, moreover minocycline on its own even in our experimental setting does not have significant analgesic activity. It would be interesting to study in patients with arthritis whether co-administration of minocycline and indomethacin (or other NSAIDs) produce better analgesic activity than indomethacin or NSAIDs alone.

The LPS-induced hyperalgesia has been suggested to be due to the release and activation of pro-inflammatory cytokines and gelatinases, as well as glial cell activation [11-15]. Minocycline inhibits the synthesis and activity of gelatinases, the synthesis and release of cytokines as well a glial activation [7,38-42]. Thus some of the analgesic activities of minocycline observed in LPS-induced hyperalgesia but not in naïve animals could be due to its activities on cytokines, gelatinases and glial cells amongst other molecules and cells which are activated and involved in pain during inflammation.

We have previously observed that higher doses of indomethacin (10 mg/kg and above) resulted in even load distribution between the arthritic right hind limb and the non-arthritic left hind limb in mice with LPS-induced arthritis similar to control animals without monoarthritis, using the Catwalk gait analysis method [10]. In the current study a lower dose of indomethacin (1 mg/kg) or minocycline at 50 mg/kg alone could not reverse the weight bearing deficits caused by monoarthritis, however, coadministration of minocycline 50 mg/kg with indomethacin at 1 mg/kg alleviated the weight bearing deficits induced by monoarthritis. The observed ability to restore weight bearing deficits has relevance to arthritis since patients with unilateral knee osteoarthritis transfer weight load from the arthritic leg to the hip and knee of the uninvolved leg; and relieving pain in the affected knee, results in even load distribution between the legs [43,44].

Minocycline has anti-inflammatory activities and is used as a disease modifying antirheumatic drug. It has been reported to inhibit T cell activation, gletanisases and other matrix proteases activity and cytokine production in rheumatoid arthritis, thus reducing inflammation [45-48]. On the other hand indomethacin is purported to produce analgesia and anti-inflammatory activity through the inhibition of cycloxygenase and prostaglandin synthesis [21,49]. Since these drugs affect different targets, all involved in inflammation and pain during arthritis, coadministration could result in an enhancement of each other's activity and be more effective at lower doses.

Other possible reasons for the enhanced antinociceptive activity by coadministering minocycline and indomethacin could have been due to the inhibition of COX enzyme activity by indomethacin and inhibition of 5-lipoxygenase activity by minocycline. The inhibition of COX-2 isoenzyme seems to be important for this synergism since coadministration of minocycline with CAY10404, a selective inhibitor of COX-2 [36] but not FR122047, a selective inhibitor of COX-1 [34,35], produced antinociceptive activity. Minocycline has been reported to block 5-lipoxygenase activation [50]. Recently, administration of a 5-lipoxygenase inhibitor has been reported to potentiate the antinociceptive activity of NSAIDs [51]. Thus, the 5-lipoxygenase inhibitory activity of minocycline could potentiate the activity of the NSAID, indomethacin. The other possible reason for the synergism on antinociception could be that the two drugs synergistically alter the endocannabinoid system in the central nervous system (CNS). Minocycline and indomethacin can both independently alter endocannabinoid levels or activity in the CNS and this has been linked to their antinociceptive activity [52,53]. Endocannabinoids have anti-inflammatory activities, antinociceptive activities and can reduce thermal hyperlagesia in rodents [54-56]. Possible biochemical pathways involved in minocycline and indomethacin enhancement of each other's antinociceptive activity warrants further research.

## Conclusions

Using mice models of inflammation-induced thermal hyperalgesia and monoarthritis we show that coadministration of minocycline with indomethacin or a selective COX-2 inhibitor, CAY10404 potentiates their effects and results in antinociception against thermal nociception, reduction of thermal hyperalgesia and alleviation of weight bearing deficits in arthritic mice at doses where either drug alone has no significant activity. Thus, the combination of lower doses of a NSAID plus minocycline could be useful in the management of inflammatory pain and arthritis with less dose-dependent side effects.

## Abbreviations

ANOVA: analysis of variance; CNS: central nervous system; i.p: intraperitoneally; LH: left hind limb; LPS: lipopolysaccharide; NSAID: non-steroidal anti-inflammatory drug; PBS: phosphate buffered saline; RH: right hind limb; S.E.M: standard error of the mean

## Competing interests

The authors declare that they have no competing interests.

## Authors' contributions

AAA participated in the acquisition and analysis of data, and helped to edit the manuscript. WM participated in the design of the study, acquisition and analysis of data, and drafting and preparation of the manuscript. Both authors read and approved the final manuscript

## Authors' details

Department of Applied Therapeutics, Faculty of Pharmacy, Kuwait University, P.O. Box 24923 Safat, 13110 Kuwait

## Pre-publication history

The pre-publication history for this paper can be accessed here:

http://www.biomedcentral.com/1471-2474/11/276/prepub
